# Intraspecific variation and symmetry of the inner-ear labyrinth in a population of wild turkeys: implications for paleontological reconstructions

**DOI:** 10.7717/peerj.7355

**Published:** 2019-07-23

**Authors:** Donald G. Cerio, Lawrence M. Witmer

**Affiliations:** 1 Department of Biological Sciences, Ohio University, Athens, OH, USA; 2 Department of Biomedical Sciences, Heritage College of Osteopathic Medicine, Ohio University, Athens, OH, USA

**Keywords:** Inner ear, Symmetry, Geometric morphometrics, Intraspecific variation, Wild turkeys, Paleontology

## Abstract

The cochlea and semicircular canals (SCCs) of the inner ear are vital neurosensory devices. There are associations between the anatomy of these sensorineural structures, their function, and the function of related biological systems, for example, hearing ability, gaze stabilization, locomotor agility, and posture. The endosseous labyrinth is frequently used as a proxy to infer the performance of the hearing and vestibular systems, locomotor abilities, and ecology of extinct species. Such fossil inferences are often based on single specimens or even a single ear, representing an entire species. To address whether a single ear is representative of a population, we used geometric morphometrics to quantitatively assess the variation in shape and symmetry in a sample of endosseous labyrinths of wild turkeys *Meleagris gallopavo* of southern Ohio. We predicted that ears would be symmetrical both within individuals and across the sample; that labyrinth shape and size would covary; that labyrinth shape would vary with the size of the brain, measured as width of the endocranium at the cerebellum; and that labyrinths would be morphologically integrated. To test these predictions, we microCT-scanned the heads of 26 cadaveric turkeys, digitally segmented their endosseous labyrinths in Avizo, and assigned 15 manual landmarks and 20 sliding semilandmarks to each digital model. Following Procrustes alignment, we conducted an analysis of bilateral symmetry, a Procrustes regression analysis for allometry and other covariates including side and replicate, and analyses of global integration and modularity. Based on Procrustes distances, no individual’s left and right ears were clearly different from each other. When comparing the ears of different specimens, statistically clear differences in shape were found in only 66 of more than 1,300 contrasts. Moreover, effects of both directional and fluctuating asymmetry were very small—generally, two orders of magnitude smaller than the variance explained by individual variation. Statistical tests disagreed on whether these asymmetric effects crossed the threshold of significance, possibly due to non-isotropic variation among landmarks. Regardless, labyrinths appeared to primarily vary in shape symmetrically. Neither labyrinth size nor endocranial width was correlated with labyrinth shape, contrary to our expectations. Finally, labyrinths were found to be moderately integrated in a global sense, but four weakly separated modules—the three SCCs and cochlea—were recovered using a maximum-likelihood analysis. The results show that both fluctuating and directional asymmetry play a larger role in shape variation than expected—but nonetheless, endosseous labyrinths are symmetrical within individuals and at the level of the population, and their shape varies symmetrically. Thus, inferences about populations, and very possibly species, may be confidently made when only a single specimen, or even a single ear, is available for study.

## Introduction

**Figure 1:**
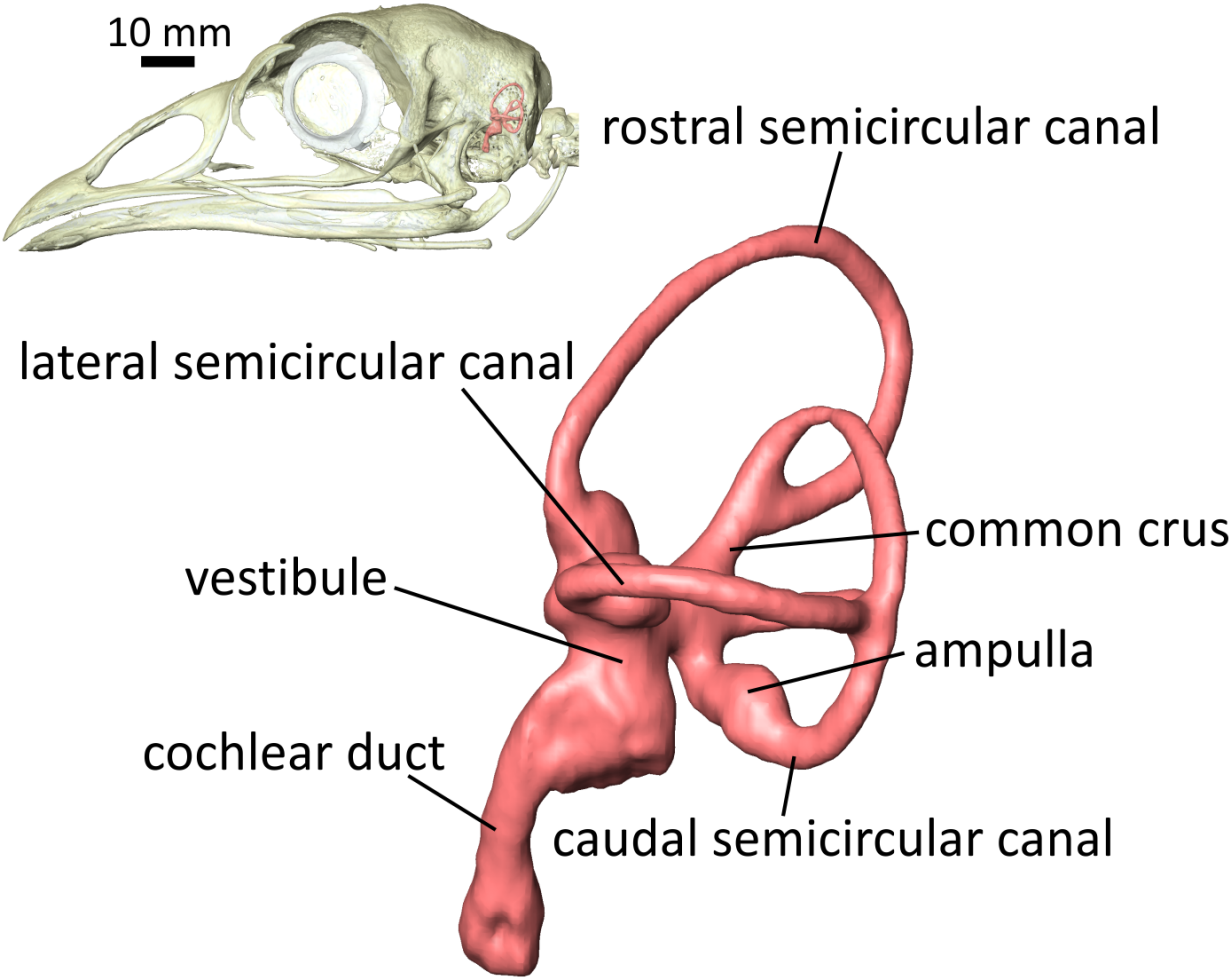
Lateral view of the left endosseous labyrinth of a wild turkey *Meleagris gallopavo*, OUVC 11410. Each labyrinth is composed of a vestibular component—the three semicircular canals (SCCs) housing the soft-tissue semicircular ducts, and the utricle and saccule within the vestibule—and a cochlear duct. The semicircular ducts sense angular acceleration, the utricle and saccule linear acceleration, and the cochlear duct sound waves. The bony signatures of these soft tissues, the endosseous labyrinth, are frequently used in paleontology to predict ranges of hearing ability and/or locomotor mode in fossil specimens of extinct species.

The soft-tissue structures of the inner ear make up critical parts of the sensorineural systems of hearing and equilibrium. The vestibular system of the inner ear—the soft-tissue semicircular ducts and their osseous housing, the semicircular canal (SCC) system (Fig. 1)—has attracted much scrutiny for its functions in gaze stabilization (Ezure & Graf, 1984; Yakushin et al., 1995; Haque & Dickman, 2005), as well as for its putative, ecomorphological connections with behavioral patterns (Spoor et al., 2007; Benson et al., 2017). The hearing apparatus of the inner ear—the cochlear duct—is also of great interest to a broad spectrum of scientists, and recent studies have found strong links between the anatomy of the cochlear duct and hearing ability (Walsh et al., 2009; Ekdale & Racicot, 2015). Since at least the mid-twentieth century, the spatial disposition of SCCs has been thought to be geometrically related to the posture of the head and neck (Duijm, 1951) and has been used to infer head posture in extinct species (Witmer et al., 2003, 2008; Hullar, 2006; Taylor, Wedel & Naish, 2009; Marugán-Lobón, Chiappe & Farke, 2013). More recently, David et al. (2010) developed a technique to reconstruct head posture and other functional parameters in extinct species by calculating the mechanical sensitivity of the vestibular system. This technique takes both spatial disposition of the bony labyrinth and physical properties of endolymph into account, and the technique was expanded upon by David et al. (2016), wherein differential staining of inner ears in computed tomographic (CT) scans was used to quantitatively assess the shape, physical properties, and mechanical sensitivity of the membranous labyrinth. Correlations have also been uncovered between the size and shape of endosseous SCCs and locomotor agility in mammals (Hullar, 2006; Spoor et al., 2007; Grohé, Lee & Flynn, 2018). A study on birds suggests that mode of locomotion may be correlated with the size of the labyrinth (Benson et al., 2017), and more work on mammals suggests that even visual acuity is related to labyrinth morphology (Kemp & Kirk, 2014). For a detailed review of the literature on morphology and function of the inner ears of mammals with respect to ecology and behavior, see Ekdale (2016).

It is clear that SCC function—specifically, the sensitivity of the ducts—is influenced by spatial disposition of the labyrinth (Dickman, 1996; Rabbitt, 1999), and evidence similarly shows correlations between hearing range and cochlear length (Walsh et al., 2009) and between radius of curvature of the cochlea and low-frequency hearing ability (Manoussaki et al., 2008). The shape of the inner ear is associated with a range of critical biological functions, and paleontologists have frequently turned to the endosseous labyrinth—which tends to fossilize well—as a basis for paleobiological inference. However, the fossil record is incomplete. Inferences of locomotor agility and head posture in extinct species are frequently based on just one specimen, possibly even just a unilateral ear, and there is a paucity of data on intraspecific variation in shape of the labyrinth. Intraspecific variation in the geometry of SCCs has been studied in some species of mammals (Haque, Angelaki & Dickman, 2004; Cox & Jeffery, 2008; Welker, Orkin & Ryan, 2009; Ekdale, 2010; Billet et al., 2012; Perier, Lebrun & Marivaux, 2016; Mennecart & Costeur, 2016; Grohé, Lee & Flynn, 2018), but many studies focus on shedding light on *inter*specific variation and include just one or a handful of specimens per species studied or do not explicitly quantify intraspecific variation (Spoor et al., 2002; Schmelzle, Sánchez-Villagra & Maier, 2007; Grohé et al., 2016). Other authors have uncovered widespread deviations from orthogonality and coplanarity among the labyrinths of diverse mammal and bird species (Malinzak, Kay & Hullar, 2012; Berlin, Kirk & Rowe, 2013; Benson et al., 2017), and there is evidence that these deviations from orthogonality and coplanarity vary widely within species (Ruf et al., 2016). Studies in mammals show that the shape of the bony labyrinth undergoes complex changes during development (Jeffery & Spoor, 2004; Ekdale, 2010). Moreover, changes in the shape of the basicranium during ontogeny have been linked to brain growth (Spoor, 1997) and to potential packing constraints involving the cerebellum (Jeffery & Spoor, 2002), although the evidence supporting hypotheses of packing constraints is mixed (Jeffery & Spoor, 2002; Jeffery, 2003).

In truth, an assessment of species- or even population-level variation (sensu Wagner & Altenberg, 1996) in the size and shape of labyrinths has not yet been undertaken in archosaurs—an assessment that could have a broad impact on the reconstruction of hearing, vestibular, visual, and locomotor abilities in the fossil record. The level of intraspecific variation in morphology of SCCs may differ among species (Billet et al., 2012; Perier, Lebrun & Marivaux, 2016; Gonzales, Malinzak & Kay, 2019), so we set out to address three major research questions or hypotheses regarding population-level variation in labyrinth shape in a single archosaur species, wild turkeys *Meleagris gallopavo*: (1) Are left and right sets of labyrinths symmetrical within an individual? Our expectation was that they would be symmetrical, based not only on the characteristic bilateral symmetry of bilaterians but also on the existence of the functional link between SCC shape and mechanical sensitivity. (2) What is the average shape of labyrinths in a population, and how symmetrical are labyrinths across that population? (3) What factors—for example, allometry—are correlated with the differences in geometry in labyrinths within a population? (4) Ultimately, can a single pair of endosseous labyrinths—or even a single left or right—serve as a proxy for an entire population? To answer these questions for wild turkeys, we employed geometric morphometrics to characterize the shape, size, and symmetry of the endosseous labyrinth and to quantify its morphological integration and modularity.

## Materials and Methods

### Turkey sample

For this study, we CT-scanned 26 intact, cadaveric heads and dry skulls of *M. gallopavo* (see Table 1 for specimen numbers and CT parameters). These were salvage specimens provided by local, permitted hunters and held in the Ohio University Vertebrate Collections (OUVC), under the terms of Permit 14-2762 issued by the Ohio Division of Wildlife. We did not collect, obtain, use, or euthanize live animals for any piece of this work. Data on sex and age were not available, but all specimens appeared to be skeletally mature based on fusion of skull sutures (Jollie, 1957). The cadaveric specimens (i.e., those with intact soft tissues) also had well-developed caruncles and wattles, and the skulls of the dry specimens were similar in size to the skulls of the soft-tissue specimens, suggesting the sample was made up entirely of adults.

**Table 1:** Specimen numbers, CT-scan parameters, and braincase dimensions of the wild turkeys in the sample.

Specimen OUVC #	Specimen nature (dry/wet)	CT scan resolution (μm)	CT scan reconstruction resolution (μm)	Width of braincase at cerebellar fossa (mm)
9803	Dry	49.3	49.3	12.0
10218	Dry	49.3	49.3	12.2
10231	Dry	49.3	49.3	12.5
10239	Dry	49.3	49.3	12.4
10443	Wet	49.3	92.0	11.8
10599	Wet	49.3	90.0	12.3
10610	Wet	49.3	90.0	11.5
10628	Dry	49.3	49.3	11.6
10657	Dry	49.3	49.3	11.7
10736	Dry	49.3	49.3	12.7
10739	Wet	49.3	90.0	12.7
10869	Dry	49.3	49.3	11.6
10884	Wet	49.3	96.7	11.4
10885	Wet	49.3	96.7	12.0
10888	Wet	49.3	96.7	12.0
10890	Wet	49.3	96.7	12.9
10891	Wet	49.3	96.7	12.6
10892	Wet	49.3	96.7	11.6
10893	Wet	49.3	96.7	12.4
11403	Dry	49.3	49.3	11.9
11406	Wet	49.3	98.6	12.7
11407	Wet	49.3	98.6	12.9
11408	Wet	49.3	98.6	12.4
11410	Wet	49.3	98.6	12.4
11411	Dry	49.3	49.3	12.2
11413	Dry	49.3	49.3	12.5

**Note:**

All specimens were scanned at a native resolution of 49.3 microns, but several scans were reconstructed at lower resolutions due to computing limitations. “Dry” refers to dried skulls, whereas “wet” refers to cadaveric heads with soft tissues intact. OUVC, Ohio University Vertebrate Collections.

Turkeys in this study were members of presumably the same population in southern Ohio, whereas the species *M. gallopavo* as a whole ranges from Mexico to Canada. Additionally, *M. gallopavo* is an old lineage, diverging more than five million years ago from its sister species *M. ocellata* (Padilla-Jacobo et al., 2018). Some populations of wild turkeys are millions of years old (Padilla-Jacobo et al., 2018), providing ample lengths of time during which morphological variation could accrue. There are pros and cons to sampling a population rather than the species across the extent of its geographic range. On the one hand, we cannot quantify shape variation across all wild turkeys in time and space with this sample alone. On the other hand, the results from studying a single population may be less likely to be confounded by evolution among geographic variants and provide a baseline framework for future studies that would sample broadly across the geographic or even temporal range of the species.

Specimens were CT-scanned at the Ohio University MicroCT Scanning Facility (OUμCT), located in Athens, Ohio. Scans were carried out with a TriFoil eXplore CT120 Small-Animal X-ray CT Scanner. Slice thickness varied slightly between scans but was not coarser than 98 microns. Resulting CT data were analyzed in Avizo Lite 9.1 (Thermo Fisher Scientific, Waltham, MA, USA) running on Dell workstations with 64-bit Windows 10 operating systems and at least 16 GB RAM. Endosseous labyrinths from each specimen were segmented and output as 3D objects (Supplemental Digital Models S1). Measurements of the skulls were also made during this process, using the native 3D Measurement tool in Avizo. The terms “endosseous labyrinth,” “inner ear,” and even simply “ear” are used as synonyms throughout.

### Shape analysis using geometric morphometrics

A total of 70 landmarks were initially used to describe the shape of 52 labyrinths—26 lefts and 26 rights (Data S1). Of the 70 initial landmarks, 10 anatomical landmarks were manually placed at the following locations: at the ventral tip of the cochlea; at the rostral-, lateral-, and caudalmost points of the lateral canal; at the rostral-, dorsal-, and caudalmost points of the rostral canal; at the dorsal- and ventralmost points of the caudal canal; and on the medial surface of the labyrinth approximately one mm ventral to the intersection of the rostral and caudal canals at the crus commune. The 60 additional semilandmarks were surface sliders, generated automatically using the “buildtemplate” and “digitsurface” commands from the R package geomorph v.3.0.7 (Adams & Otárola-Castillo, 2013) in R v.3.5.1 (R Core Team, 2018). The “buildtemplate” command uses a nearest-neighbor algorithm to generate a template of these approximately equidistant semilandmarks. These semilandmarks do not slide along curve tangents but instead slide on surface tangents, minimizing bending energy, when the template is applied to each specimen using “digitsurface” (algorithm described in Gunz, Mitteroecker & Bookstein (2005) and Mitteroecker & Gunz (2009)).

**Figure 2:**
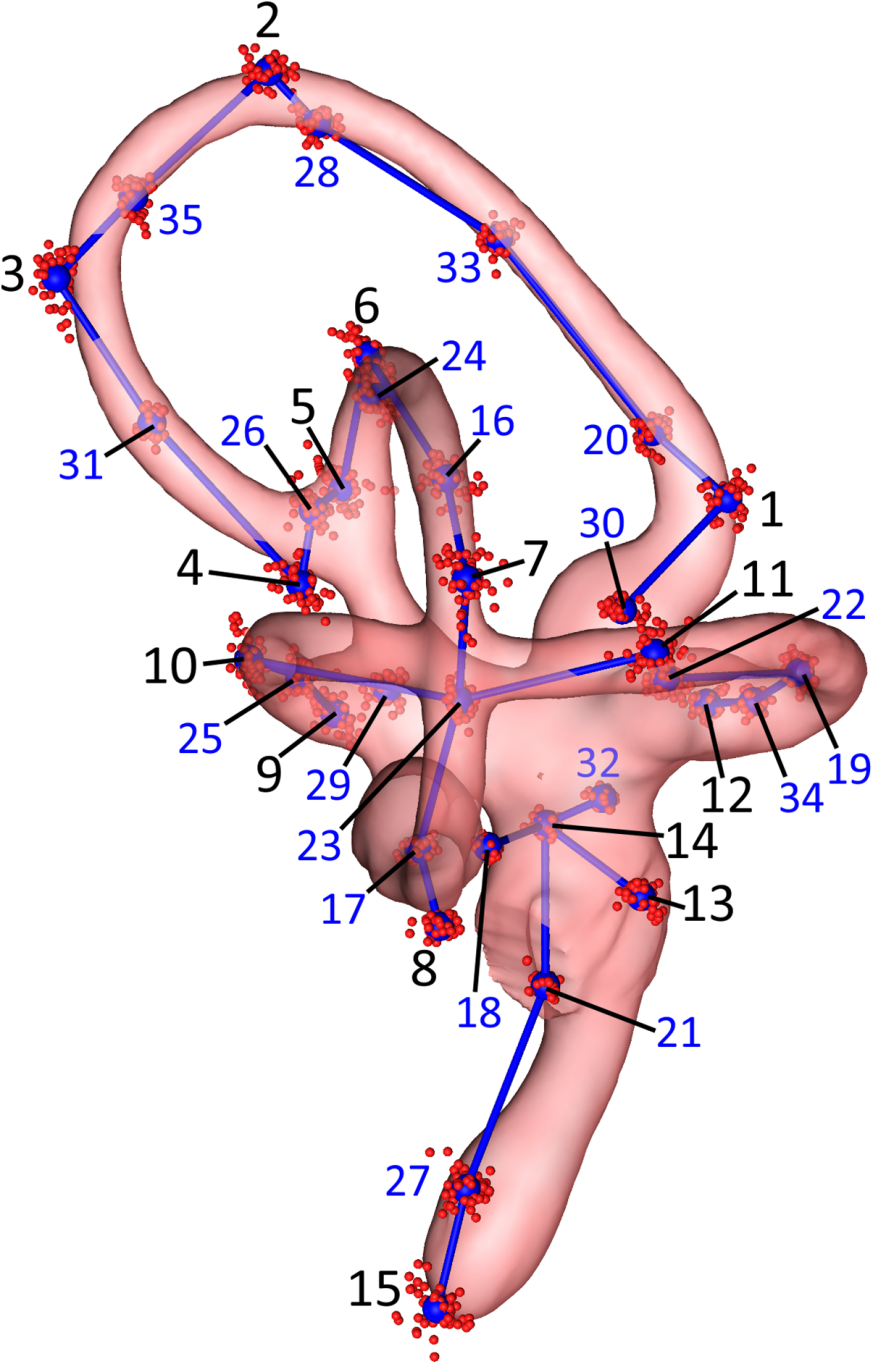
Lateral view of the landmarks from all 26 turkey specimens following Procrustes alignment. The right endosseous labyrinth of turkey OUVC 11408 is transparent, scaled, and superimposed for reference. Red point-clusters symbolize the landmarks from individual turkeys, while the larger blue points within the clusters represent mean positions of each landmark. A set of blue lines connect the mean points for additional spatial context. The black numbers 1–15 indicate the landmarks that were manually assigned: landmarks 1–4 for the rostral canal, 5–8 for the caudal canal, 9–12 for the lateral canal, and 13–15 for the cochlea and vestibule. The blue numbers 16–35 indicate the landmarks that were placed automatically using the command “digitsurface” from the R package, geomorph. More detailed information on landmark location may be found in Table 2.

We then performed a subsampling analysis of these 70 initial landmarks using the “lasec” command from the R package LaMBDA (LandMark-Based Data Assessment) v.0.1.0.9 (Watanabe, 2018). The subsampling analysis indicated that 35 landmarks were sufficient to describe the shape of the inner ears in the sample. A total of 15 of these were manually placed, anatomical landmarks, digitized using the “digitsurface” command from the R package geomorph v.3.0.7 (Adams & Otárola-Castillo, 2013), and 20 were surface sliders (Fig. 2; Table 2) automatically generated using the “buildtemplate” and “digitsurface” commands as described above. We increased the number of anatomical landmarks from 10, in the initial set of 70 landmarks, to 15, in the empirically reduced set of 35 landmarks, to more evenly distribute these manually placed landmarks across each of the SCCs and the cochlea. The R code used for the subsequent analyses of these data is in Data S2. Landmarks were digitized twice for each ear, for a total of four replicates—two lefts and two rights—per specimen. For the purposes of comparison, landmarks from right-side labyrinths were reflected by multiplying the *Z*-coordinates of the landmarks by −1 to yield 104 total sets of “left” ears (Data S3).

**Table 2:** Procrustes variances of each landmark in the analysis.

Landmarks		Location	Procrustes variance
Number	Type		
Rostral canal			
1	Anatomical	Rostralmost point	0.229
2	Anatomical	Dorsalmost point	0.871
3	Anatomical	Caudalmost point	0.198
4	Anatomical	Just caudoventral to intersection between rostral canal and common crus	0.00285
20	Surface slider	Just caudodorsal to 1	0.296
28	Surface slider	Just ventral to 2	0.748
30	Surface slider	Ampulla of canal	0.0509
31	Surface slider	Rostroventral to 3, halfway between 3 and 4	0.0548
33	Surface slider	Rostroventral to 2, halfway between 1 and 2	0.618
35	Surface slider	Just rostrodorsal to 3	0.391
Caudal canal			
5	Anatomical	Just medial of intersection between caudal canal and common crus	0.0523
6	Anatomical	Dorsalmost point	0.226
7	Anatomical	Lateralmost point	0.399
8	Anatomical	Ventralmost point	0.447
16	Surface slider	Just dorsomedial to 7	0.294
17	Surface slider	Just dorsal to 8	0.284
23	Surface slider	Just ventromedial to 7	0.284
24	Surface slider	Just ventral to 6	0.168
26	Surface slider	Just caudoventral to 5, at intersection of rostral and caudal canals	0.0201
Lateral canal			
9	Anatomical	Caudomedialmost point, at intersection of lateral canal and common crus	0.0776
10	Anatomical	Caudalmost point	0.141
11	Anatomical	Lateralmost point	0.358
12	Anatomical	Ampulla of canal	0.0694
19	Surface slider	Rostromedial to 11, halfway between 11 and 12	0.0538
22	Surface slider	Just medial to 11	0.243
25	Surface slider	Just rostral to 10	0.0970
34	Surface slider	Lateral to 12, halfway between 12 and 19	0.00335
Cochlea and vestibule			
13	Anatomical	Midpoint of oval window	0.228
14	Anatomical	Medial face of labyrinth, at crease between vestibule and canals	0.193
15	Anatomical	Ventralmost point of cochlear duct	2.05
18	Surface slider	Caudal contour of vestibule, near caudodorsal edge of round window	0.166
21	Surface slider	Ventromedial to oval window on lateral face of cochlear duct	0.558
27	Surface slider	Just rostrodorsal to 15, on rostral face of cochlear duct	1.50
29	Surface slider	Lateral to 9, on lateral face of vestibule	0.0322
32	Surface slider	Rostrolateral to 14 and rostroventrolateral to 12, on rostral face of vestibule	0.164

**Note:**

These are grouped by the four likeliest modules from the EMMLi analysis. The most variable landmarks by far were landmarks 15 and 27, the two nearest the tip of the cochlear duct. After these, the landmarks nearest the dorsalmost point of the rostral canal were most variable.

Our first hypothesis, H1_0_, was that ears would be bilaterally symmetrical both within individuals and within the sample. The first alternate hypothesis, H1_A_, was that ears would be asymmetrical either within individuals, within the sample, or both. We tested this set of hypotheses by conducting an analysis of directional and fluctuating asymmetry, first conducting in R a generalized Procrustes analysis (Rohlf & Slice, 1990) using the command “gpagen,” followed by an analysis of bilateral symmetry (Mardia, Bookstein & Moreton, 2000; Klingenberg, Barluenga & Meyer, 2002) using the command “bilat.symmetry.” Landmarks were aligned so as to minimize bending energy. We also ran an analysis of variance (ANOVA) of Procrustes coordinates with side using the command “procD.lm,” and significance testing was carried out using 4,999 iterations. To visualize similarities and differences between the mean left and mean right labyrinths, we also used the software CloudCompare v.2.10.2 (www.cloudcompare.org). Three-dimensional meshes of the mean left, mean right, and sample-mean ears were first created in R by warping the labyrinth of specimen OUVC 11408 using the command “plotReftoTarget” from the package geomorph. This specimen’s labyrinth was chosen because it was determined to be nearest the mean shape by the command “mshape,” again from the package geomorph. Models of the mean left, mean right, and sample-mean ears were then exported using the command “writeSTL” from the package rgl (Adler, Murdoch & others, 2018), and then these models were imported to CloudCompare. Using the “Cloud/Mesh dist” tool in this program, distances in millimeters between vertices of the mean left and sample-mean ear models, as well as between the mean right and sample-mean models, were computed. These distances were projected onto the mean left and mean right ear models as heat maps.

Our second hypothesis, regarding the overall allometry of inner ears, was split into two sets of sub-hypotheses. First, we hypothesized that there would be an allometric relationship between the shape and size of inner ears, with size measured as centroid size (H2i_A_). The corresponding null hypothesis was that the shape and size of inner ears in the sample would not covary (H2i_0_). The second sub-hypothesis was that the allometric relationship between labyrinth shape and labyrinth size would not be different for left and right ears (H2ii_0_). The alternative, H2ii_A_, was that there would be different, or asymmetric, allometric relationships between these two quantities. We tested these hypotheses by running regression analyses with the command “procD.allometry.” In these regressions, we treated labyrinth shape as the response variable and centroid size as the explanatory variable. Significance testing was carried out using 4,999 iterations.

Our third hypothesis, H3_A_, was that the shape of inner ears will covary primarily with ear size, brain size, measurement error, and/or interactions between these terms. The null hypothesis, H3_0_, was then that the shape of labyrinths will not covary predictably with these terms. As the cerebellum occupies the endocranial space between the left and right labyrinths, we expected that ear shape would covary with size of the brain, and particularly with size of the cerebellum. We measured width of the endocranial cavity at the cerebellum as a proxy for the brain size of the turkey specimens in our sample, using the 3D-measurement tool in Avizo. We tested this third pair of hypotheses using linear regressions carried out using the command “procD.lm,” again with significance testing carried out using 4,999 iterations.

### Integration/Modularity

Recent studies have found patterns of strong morphological integration in bird skulls when examining a limited range of species (Stange et al., 2018), whereas others have found patterns of modularity and mosaic evolution across a broad, deep sample of avian phylogeny (Felice & Goswami, 2018). Even in the latter studies, however, integration and constraint tend to be recovered in regions of the skull derived from single lineages of cell types, especially from mesoderm and from posterior-mandibular cranial neural-crest cells (Felice & Goswami, 2018).

Although the development of the inner ear involves signaling factors from several tissue types (Park & Saint-Jeannet, 2008), the pattern and timing of developmental events seems to be remarkably conserved across vertebrates (Torres & Giráldez, 1998). We hypothesized that the overall trend in the shape of inner ears will be one of integration (H4i_0_), with an alternate hypothesis of overall disintegration (H4i_A_). We tested these hypotheses using the “globalIntegration” command from the geomorph package in R. This function quantifies the bending energies of Procrustes-aligned sets of landmarks at different spatial scales. The function plots the log-transformed bending energies against the log-transformed variance of the partial warps of the aligned sets of landmarks (Bookstein, 2015). A regression of these data is then calculated by the function. If the slope of this regression is steeper than −1 (i.e., is more negative), the data are consistent with a hypothesis of overall integration. If the slope is more shallow (i.e., between −1 and 0), the pattern would be consistent with data that are disintegrated (Bookstein, 2015).

Although the development of the vertebrate inner ear seems to be highly conserved, the two major divisions of the inner ear, the cochlear and semicircular ducts, serve disparate functions (for a review of the functions of the two divisions of the inner ear, see Ekdale, 2016). Furthermore, the three ipsilateral semicircular ducts are functionally linked to different muscles in the orbit via different sets of cranial nerves (Yakushin et al., 1995). We hypothesized that SCCs could be morphologically modular with respect to each other and to the cochlear duct (H4ii_A_), with the null hypothesis being overall morphological integration (H4ii_0_). We tested these hypotheses using the “EMMLi” command in the EMMLi (evaluating modularity with maximum likelihood) package v.0.0.3 (Goswami & Finarelli, 2016) in R. This function uses a maximum likelihood approach to compare the trait correlation matrices of competing models of modularity and integration. These models are compared using log-likelihoods and the Akaike Information Criterion, and this technique is robust to large variations in the structure and complexity of the hypothesized modules (Goswami & Finarelli, 2016). We tested the following four models, with varying numbers of modules: (A) single module, complete integration; (B) two modules, cochlea+vestibule vs. SCCs as a whole; (C) four modules, lateral canal vs. rostral canal vs. caudal canal vs. cochlea+vestibule; and (D) five modules, lateral canal vs. rostral canal vs. caudal canal vs. cochlea vs. vestibule.

We chose to conduct the set of analyses relating to H4ii using the EMMLi package because this package was able to accurately identify the correct number of parameters and strength of integration, following Procrustes alignment, using simulated data under a broad range of conditions in testing by Goswami & Finarelli (2016). Recent evidence suggests that analyses of morphological integration may return spurious results when applied to Procrustes-aligned shape data and when allowing semilandmarks to slide (Cardini, 2019). The EMMLi statistical package was not among the group of statistical methods tested by Cardini (2019) for accuracy. However, as a cautionary step, we conducted another set of analyses to address the potential for spurious results in our initial test of H4ii. In this second set of analyses, we conducted separate Procrustes alignments on the landmarks in each of the modules defined in the most likely model returned by the EMMLi analysis. Then we tested the degree of morphological integration in each module individually, using the “globalIntegration” test, as in our earlier analysis of the overall integration within the sample (H4i). Finally, the amount of variation within modules was assessed with the command “morphol.disparity,” in a workflow similar to that of Stange et al. (2018).

## Results

Note: Following the recommendations of Dushoff, Kain & Bolker (2019) regarding longstanding problems with the term “statistical significance,” we often opt for interpreting the outcome of statistical analyses as being “clear” or “unclear” and citing the relevant quantitative measures (e.g., *p*-values, Sum of Squares [SS]).

### Initial alignment and analysis of asymmetry

Following the Procrustes alignment, the initial analysis of bilateral symmetry found that components of both directional asymmetry (*p* = 0.002, SS = 0.0037, effect size [*Z*] = 2.54) and fluctuating asymmetry (*p* = 0.002, SS = 0.043, *Z* = 28.2) in regards to shape variation were clearly present in the dataset. However, both effects appeared to be much smaller than individual variation (*p* = 0.002, SS = 0.260, *Z* = 5.51) and smaller than measurement error (SS = 0.049), itself a minor component of variance. Centroid size also appeared to vary between left and right sides on average (*p* = 0.002, SS = 0.076, *Z* = 1.07) and within individuals (*p* = 0.002, SS = 0.530, *Z* = 18.7), but these effects were several orders of magnitude smaller than that of individual variation (*p* = 0.002, SS = 65.8, *Z* = 1.29). Measurement error was again a minor component of variance (SS = 0.330). Additionally, when we conducted a Procrustes ANOVA with the “procD.lm” command, the relationship between shape and side was not statistically clear (*p* = 0.89, SS = 0.0018, *Z* = −1.29). These two separate statistical tests produced starkly different results, but either way, the sums of squares indicated that the effects of asymmetry in this dataset were small.

**Figure 3:**
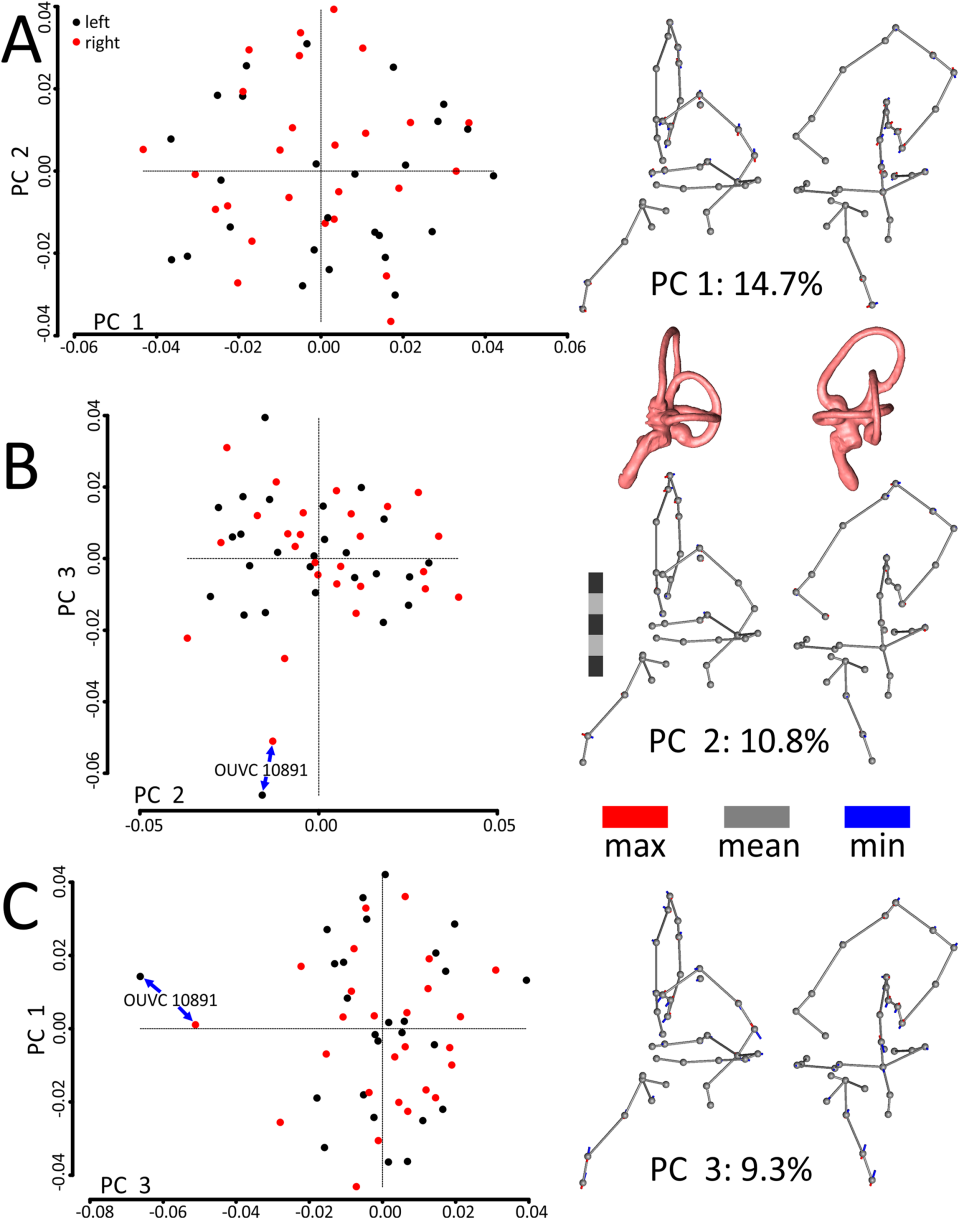
Scatterplots and ball-and-stick visualizations of the first three principal components (PCs) of the variation in shape of turkey labyrinths. The first scatterplot (A) shows PC1 and PC2, (B) shows PC2 and PC3, and (C) shows PC1 and PC3. Scatterplots are flanked by ball-and-stick visualizations, in rostrolateral and caudolateral views, which show the differences between minimum and maximum scores of PC1 (A), PC2 (B), and PC3 (C). The labyrinths inset between the PC1 and PC2 ball-and-stick visualizations are also in rostrolateral and caudolateral views, for reference. The scale bar is five mm. In the scatterplots, left ears are represented by black points and right ears are represented by red points. In the ball-and-stick models, the gray lines represent the shape of the sample-mean ear, with red and blue sticks representing maximum and minimum scores, respectively, on the PC axes. For example, if an ear scored positively on PC1, the ventral part of its cochlea was tilted medially relative to the species mean, whereas its rostral canal was tilted laterally relative to the species mean. Conversely, if an ear scored negatively on PC1, its cochlea was tilted laterally relative to the mean, and its rostral canal tilted medially relative to the mean. As evidenced by the small red and blue sticks in the ball-and-stick visualizations, shape differences in this sample were generally miniscule. Right and left ears from the same individual were indistinguishable from each other, across each of the 26 turkey specimens, when using Procrustes distances to conduct statistical contrasts. The left labyrinth of OUVC 10891 appeared to be separated from the dataset on PC3. Nevertheless, this labyrinth clustered near its right mate, which itself was not clearly statistically different from other ears.

A principal components (PCs) analysis applied to the Procrustes-aligned coordinates revealed that the first principal component (PC1), which explained only about 15% of the overall variation in shape, related almost entirely to mediolateral tilt of the cochlear duct, rostral canal, and caudal canal (Fig. 3; see Fig. S1 for another view of the PC scatterplots with pairs of ears labeled and color-coded by specimen). The second principal component (PC2), which explained about 11% of the overall variation in shape, primarily related to the height and rostral protrusion of the rostral canal, as well as the rostrocaudal position of the SCCs as a unit relative to the cochlear duct. The third principal component (PC3), which explained about 9% of the overall variation in shape, primarily related to the overall height of the labyrinth. Broadly speaking, the main geometrical difference among labyrinths in this dataset concerns the relative orientation of SCCs and cochlea, with a greater angle separating the SCCs and the cochlea at minimum values of PC1. In total, five PCs encompassed approximately 50% of the total variation in shape, but it took 21 PCs to encompass 90% and 85 to encompass >99.9%.

**Figure 4:**
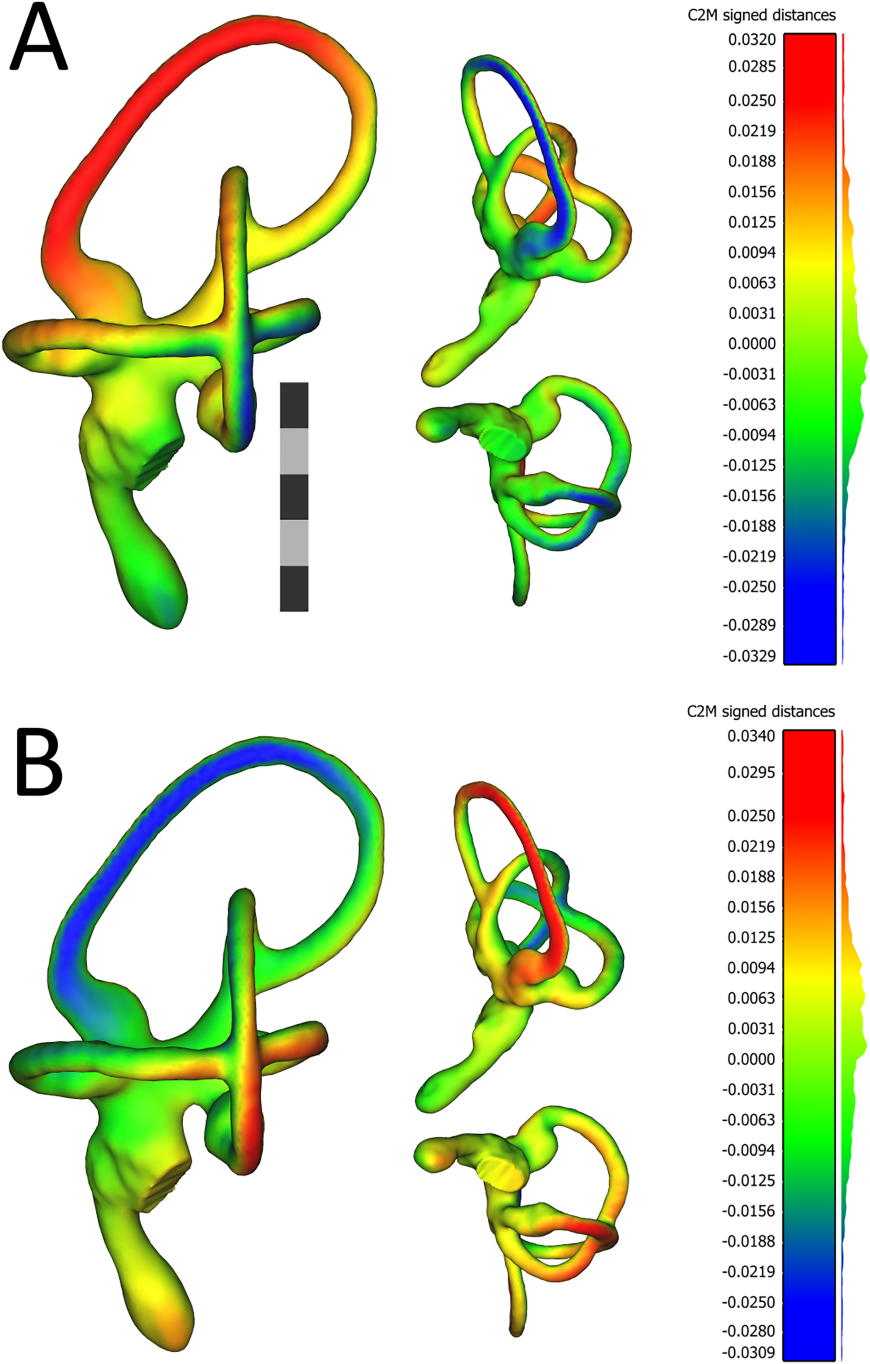
Views of 3D models of the mean left (A) and mean right (B) ears from the sample of wild turkeys. The scale bar is five mm. In both (A) and (B), models are presented in caudolateral (left), rostrodorsal (top-middle), and ventral (bottom-middle) views. The heat maps on the models correspond to the histograms on the right, which indicate distances in millimeters between the vertices of the ear in question and the corresponding vertices of the sample mean ear. The rostral canal of the mean left ear appears to be angled very slightly more acutely, relative to the lateral canal (i.e., it is tilted more laterally), than the rostral canal of the right ear. The red color in the heat map in (A) indicates the lateral tilt, by about 30 μm, of the rostral canal relative to the sample mean ear. In (B), the blue color on the rostral canal of the mean right ear indicates this canal is tilted slightly medially, again by about 30 μm, relative to the sample mean ear. As indicated by the histograms, the bulk of the vertices in the mean ears of each side are fewer than 12 μm distant from the corresponding vertices in the sample mean ear. Images of the models, as well as the histograms, were produced in CloudCompare v.2.10.2 as detailed in the section “Shape Analysis using Geometric Morphometrics.”

The first PC of asymmetric shape variation (∼16%), or the primary axis on which left ears differed from right ears across the dataset, related to several small geometrical differences: (A) the rostrocaudal deviation of the dorsal half of the caudal canal; (B) the caudal extension of the caudal half of the rostral canal; (C) the dorsoventral deviation of the rostral half of the lateral canal; and (D) degree of torsion of the cochlear duct. See Fig. 4 for a visualization of the differences between the sample-mean left and sample-mean right ears. The first PC of symmetric shape variation (∼16%), or the primary axis on which left and right ears *as a unit within specimens* varied across the dataset, closely resembled PC1 of overall shape variation.

### Allometric effects

In separate analyses, both centroid size (*p* = 0.0014, SS = 0.008, *Z* = 2.76) and width of the endocranium at the cerebellum (*p* = 0.0112, SS = 0.0069, *Z* = 2.19) were found to be associated with shape of the labyrinth—but only if we treated different replicates as separate ears. Since this effectively was an artificial inflation of the sample size, we took the mean of each labyrinth’s replicate measurements to yield mean left and right ears for each specimen. When we conducted allometric regressions with these mean shapes, neither centroid size (*p* = 0.136, SS = 0.0041, *Z* = 1.08) nor endocranial width (*p* = 0.301, SS = 0.0034, *Z* = 0.539) fell out clearly as predictor variables. Additionally, predictive power of the models was extremely low, with an *R*^2^ for centroid size of 0.022 and an *R*^2^ for endocranial width of 0.019. The predicted “max” and “min” shapes were visually indistinguishable, and slopes were not different between left and right ears. Ultimately, we could not reject H2i_0_ or H2ii_0_.

A clear relationship was recovered between shape of the labyrinth and an interaction effect between the size of the centroid of the labyrinth and endocranial width, but this relationship explained little variation (SS = 0.008, *Z* = 3.32) relative to the residuals (SS = 0.137) and it had a weak coefficient of determination (*R*^2^ = 0.051). Taken altogether, although we can reject H3_0_, the relationship between shape of ears of adult turkeys and the size metrics in this study is not predictive. Additionally, no relationship between measurement error and shape was recovered.

### Integration/Modularity

We found ears to be somewhat globally integrated. When the log-transformed bending energies of the Procrustes-aligned landmarks were regressed against the log-transformed partial warps, the resulting slope was −1.29. This was near but slightly steeper than −1, so while we were unable to reject H4i_0_ of global integration of the landmarks in this sample, the integration appeared to be weak. Furthermore, an EMMli model with four modules, representing the lateral SCC, rostral canal, caudal canal, and cochlea+vestibule, had the highest posterior probability (0.427) and second-best log-likelihood (657), allowing us to reject H4ii_0_. The within-module integration in this model was moderate, with a ρ score of 0.320, and was the same in every module. The between-module integration was present but not very strong, with a ρ score of 0.170, and also the same between each pair of modules. Four additional models were recovered with posterior probabilities greater than 0.05. Two of these models shared the four-module structure of the most likely model but had different patterns of between- and within-module integration, while the other two models included five modules. A list of all models with posterior probabilities greater than 0.05, including maximum likelihoods and values of ρ, is available in Table 3.

**Table 3:** Results of the EMMLi analysis of morphological integration.

Model	Log-likelihood	Posterior probability	ρ within modules	ρ between modules
Four modules	657	0.427	0.320	0.170
Same ρ between and within modules				
Four modules (different ρ within modules)	658	0.0520		0.170
Rostral canal			0.320	
Caudal canal			0.320	
Lateral canal			0.280	
Cochlea+vestibule			0.350	
Four modules (different ρ between modules)	662	0.225	0.320	
Rostral—caudal canals				0.190
Rostral—lateral canals				0.140
Caudal—lateral canals				0.170
Rostral canal—cochlea+vestibule				0.140
Caudal canal—cochlea+vestibule				0.210
Lateral canal—cochlea+vestibule				0.140
Five modules	656	0.0965	0.320	0.170
Same ρ between and within modules				
Five modules (different ρ within modules)	660	0.142		0.170
Rostral canal			0.320	
Caudal canal			0.320	
Lateral canal			0.280	
Cochlear duct			0.390	
Vestibule			0.510	

**Note:**

The five most-supported models of integration are detailed here. Each model bears similarities to the others, including low amounts of integration between modules and low-to-moderate integration within modules. In the five-module model with different amounts of integration within models, there is some evidence that the cochlear duct and vestibule are more highly morphologically integrated than the other three modules. EMMLi, evaluating modularity with maximum likelihood; ρ, rho, optimal estimate of the correlation coefficient for a given module or set of modules where 0.99 represents very strong integration and 0.01 represents very weak integration.

When the integration of each of the canals and cochlea+vestibule was tested using the “globalIntegration” command, a pattern similar to the results of the EMMLi analysis was recovered. The slope of a regression between the log-transformed bending energy and log-transformed partial warps of landmarks in the rostral canal was −1.50, clearly steeper than −1 and a signal of integration within this module. The slope of this regression for the cochlea+vestibule was similar at −1.72, again a signal of integration within this module. Slopes for the lateral and caudal canals were approximately equal to −1, indicating self-similarity (sensu Bookstein, 2015) rather than integration or disintegration. Combinations of the four modules were also tested for signs of integration between landmarks of different modules (Table 4). In general, regressions between the bending energy and partial warps of these combinations yielded slopes approximately equal to −1 or slightly steeper than −1, indicating self-similarity or weak integration. The exception to this was the combined set of landmarks from the lateral canal and cochlea+vestibule, which had a regression slope of −0.735, indicating some disintegration between the landmarks of these two modules.

**Table 4:** Results of “globalIntegration” tests on individual modules and combinations of modules, and comparison with EMMLi results.

Module	Slope of BE—PW regression	Consistent with EMMLi?
Rostral canal	−1.50	Yes
Caudal canal	−0.971	No
Lateral canal	−1.02	Unclear
Cochlea+vestibule	−1.72	Yes
Rostral—caudal canals	−0.992	Yes
Rostral—lateral canals	−1.03	Yes
Caudal—lateral canals	−1.18	Yes
Rostral canal—cochlea+vestibule	−1.06	Yes
Caudal canal—cochlea+vestibule	−1.10	Yes
Lateral canal—cochlea+vestibule	−0.735	Yes
Rostral—caudal—lateral canals	−1.22	Yes

**Note:**

In general, the “globalIntegration” analyses on individual modules and combinations of modules found patterns of self-similarity or weak-to-moderate integration within modules. Consistent with the results from the EMMLi analysis, the modules representing the rostral semicircular canal and cochlea+vestibule showed evidence of more integration than the lateral canal. Landmarks of the caudal canal did not appear to show a pattern of integration but also did not appear to be disintegrated. Landmarks of the lateral canal similarly did not show a pattern of integration, but the EMMLi analysis did result in some evidence for a weaker pattern of integration in this module, so the two analyses may be consistent. When combining landmarks from two or more modules, the “globalIntegration” analysis generally returned patterns of weak integration or disintegration. These results are broadly consistent with results of the EMMLi analysis. BE—PW, log-transformed bending energy and log-transformed partial warps; EMMLi, evaluating modularity with maximum likelihood.

### Morphological disparity

In all, the turkey ears in this sample showed relatively little variation, with an overall Procrustes variance (PV) of 0.00291. Tellingly, an analysis of morphological disparity found that none of the left and right ears within each individual turkey could be statistically differentiated from each other based on PV. When we compared all 52 labyrinths against each other using PV, statistically clear differences were found in just 66 of the possible 1,326 contrasts. Additionally, 43 of these 66 contrasts involved a single ear—the left labyrinth of OUVC 10891 (which was not statistically different from its right mate). If we remove that ear from consideration, differences between ears were statistically clear in just 23 out of 1,275 contrasts, meaning that labyrinths were not differentiable by PV in more than 98% of the contrasts.

When we broke down PV by module identified in the section “Integration/Modularity” and then corrected for the number of landmarks within each module (as in Stange et al., 2018), the caudal canal showed the most variance, 0.0000966, across nine landmarks. The rostral canal showed the next-most variance, 0.0000918, across 10 landmarks; the cochlea+vestibule showed the third-most variance, 0.0000769, across eight landmarks; and the lateral canal showed the least variance, 0.0000738, across eight landmarks. Uncorrected PV of individual landmarks are detailed in Table 2. The overall median PV for manually placed, anatomical landmarks was 0.226 (*n* = 15, mean = 0.369, s.d. = 0.511), whereas the median PV for sliding semilandmarks was 0.205 (*n* = 20, mean = 0.301, s.d. = 0.35). A post hoc Welch’s *t*-test, performed in R, did not recover a clear difference in the mean PV of anatomical and sliding semilandmarks (d.f. = 23.4, *t* = 0.443, *p* = 0.66).

## Discussion

### Variation in the shape of labyrinths in a population of turkeys is low

The amount of overall morphological disparity within the sample was low, as was the morphological disparity within each module of the labyrinths, which was evidenced by the inability to statistically differentiate ears by Procrustes distance, as discussed in the section “Morphological Disparity.” However, effects of asymmetry on shape were clearly recovered within this population. Before discussing potential causes and plausible implications of this, it is worthwhile to briefly review the mathematics of symmetry within samples. Whereas symmetry is a central tendency with regard to fluctuating asymmetry, this is not the case with regard to directional asymmetry, where the state of being symmetric is a wall. In other words, while a structure or set of structures that appear to be symmetric might vary in shape in a bell curve around a sample mean—that is, fluctuating asymmetry—that mean cannot represent a state of symmetry “greater” than the state of symmetry itself (Van Valen, 1962; Palmer & Strobeck, 1986; Klingenberg, 2015). Mathematically, a structure or set of structures show perfect directional symmetry in shape across a population only if the difference in mean shape between them is zero; any deviation from this signifies directional asymmetry (Klingenberg, 2015). Perfect directional symmetry is a mathematical ideal (Van Valen, 1962).

This result, then, may be an artifact derived from confounding sources—perhaps most importantly, the number of free variables, 105, exceeding the number of specimens, 26, which can adversely affect statistical power (Gunz & Mitteroecker, 2013; Collyer, Sekora & Adams, 2015). Still, the number of landmarks appears to be appropriate to describe the shape of the labyrinth, based on empirical testing using a recently published R package (Watanabe, 2018). It is also possible that landmark placement was biased, but Procrustes coordinates did not clearly covary with replicate; that is, measurement error did not appear to be a factor. It is possible that the individuals in the sample had ears that were more asymmetric than the population at large, and perhaps a larger sample would have resulted in a different set of results with regard to fluctuating asymmetry (Babbitt, Kiltie & Bolker, 2006). Larger samples are certainly always ideal, but this was a fairly robust sample (*N* = 26) that met the recommended sample size by Cardini, Seetah & Barker (2015) of greater than 20 specimens for estimation of mean shape and no fewer than 15–20 specimens for estimation of size and shape variance. The sample of turkeys in this study was comparable to but larger than the sample in a similar study on the inner ears of ruminants (Mennecart & Costeur, 2016). Finally, a key assumption of Procrustes ANOVA is that landmarks vary in their coordinates equally and isotropically (Klingenberg, Barluenga & Meyer, 2002), an assumption that in biological datasets is unlikely (Klingenberg & McIntyre, 1998). Indeed, the landmarks in this dataset clearly violate this assumption, with landmarks on the ventral cochlea and on the dorsalmost curve of the rostral canal being at least an order of magnitude more variable than most of the remaining landmarks. In cases like these, mean squares and sums of squares themselves might be more appropriate measures of effects (Klingenberg, Barluenga & Meyer, 2002)—and in the present study, these quantities were generally much smaller than the residuals.

It is also plausible that the directional asymmetry recovered in this analysis is real—but small and not biologically (e.g., physiologically) relevant. The variance associated with side, relative to the variance associated with individual and with measurement error, was very small. When plotted using 3D-visualization tools (Fig. 4), the “mean” configurations of left and right labyrinths are virtually identical. It is entirely possible that even if these small deviations from symmetry do affect afferent signals to the brain, the brain may be able to “do the math” and correct for them.

A final possibility is that this directional asymmetry is both real and physiologically relevant. Perhaps a larger sample would be perfectly bilaterally symmetrical on the average, but maybe turkeys are more variable than more aerobatic birds. A variety of studies have uncovered relationships between the geometry of SCCs and locomotor or visual abilities (Spoor et al., 2007; Jeffery & Cox, 2010; Kemp & Kirk, 2014; Benson et al., 2017), and others have found physiological relationships between these systems (Haque, Angelaki & Dickman, 2004; Haque & Dickman, 2005). Given that turkeys are generally herbivorous ground birds rather than, say, aerial pursuit predators, their inner ears may be less constrained (under less intense stabilizing selection), opening up individuals and populations to be more asymmetric. Indeed, recent work has shown that in some mammal groups, including sloths and primates, slower-moving species display higher intraspecific variation in the shape of SCCs than do faster-moving relatives (Billet et al., 2012; Perier, Lebrun & Marivaux, 2016; Gonzales, Malinzak & Kay, 2019).

Previous researchers have uncovered robust interspecific relationships between the size and shape of the labyrinth itself (Lebrun et al., 2010; Billet, Hautier & Lebrun, 2015) as well as between body size and labyrinth shape (Alloing-Séguier et al., 2013; Neenan et al., 2017) and between body size and centroid size of the labyrinth (Benson et al., 2017). In the present study on turkeys, shape and size of inner ears did not seem to covary, contrary to what was expected. This lack of a relationship did not appear to differ by side. The coefficient of determination was extraordinarily weak and non-predictive. This same pattern was found with an endocranial metric of size, suggesting that if there are packing constraints involved in the shape of labyrinths in adult turkeys, they are weak constraints. Indeed, the only covariate of shape variation that had any predictive power was individual identity of the turkey. The fact that shape and size of inner ears did not clearly covary in this sample of turkeys makes some sense when considering that all specimens were of adult size. Ontogenetic work in the opossum *Caluromys* has found that the height of the rostral and caudal canals relative to the common crus changes through development (Sánchez-Villagra & Schmelzle, 2007), and work in humans has shown that the angular relationships between rostral and caudal canals change throughout development (Jeffery & Spoor, 2004), but other aspects of shape such as canal radii do not appear to change and few changes in shape occur at all once ossification is complete (Jeffery & Spoor, 2004). Similarly, in an ontogenetic study in *Monodelphis*, Ekdale (2010) found that although the overall size of the labyrinth was correlated with length of the skull, labyrinth shape did not clearly covary with age in this taxon after the completion of ossification.

### Morphological integration vs. modularity

Based on the results from the analyses of morphological integration, which found that the inner ears of turkeys are weakly-to-moderately integrated in a global sense, we cannot reject the null hypothesis of global integration (H4i_0_). However, we do reject the null hypothesis of overall integration between the lateral canal, rostral canal, caudal canal, and cochlear duct (H4ii_0_) based on the results of the EMMLi analysis of integration, which returned evidence that each SCC and the cochlea+vestibule are somewhat modular with respect to each other. Still, the level of modularity of inner ears does not appear to be complete, either, as the EMMLi analysis also found only moderate levels of within-module integration.

Although finding both integration and modularity might seem contradictory, these results make sense in the context of the development and function of the inner ear. Torres & Giráldez (1998) found that, across vertebrates, the pattern and timing of developmental events of inner ears are conserved, and recent studies have found that the shape of inner ears does not change much with age once the skull has ossified (Jeffery & Spoor, 2004; Ekdale, 2010). In humans, the angle between the cochlea and the SCCs, as well as the torsion of the rostral canal, continues to change after ossification but the changes are small (Jeffery & Spoor, 2004). In the present study, the principal axis of shape variation in the inner ears of turkeys also relates largely to the tilt of the rostral canal and cochlear duct, but differences across the dataset are small, echoing the result from Jeffery & Spoor (2004). It may be that once the otic capsule is fully ossified, any subsequent morphological changes to the dense bone in the temporal region would affect the entire labyrinth as a unit. Still, each SCC seems to be a coherent morphological module—albeit with only a moderate degree of within-module integration—which may reflect differences in functional roles in the vestibuloocular reflex (Yakushin et al., 1995) or the complex shape changes that occur prior to labyrinths reaching adult size (Jeffery & Spoor, 2004; Ekdale, 2010).

It is worth repeating here that there is recent evidence suggesting that studies of morphological integration can return spurious results when employing global Procrustes alignments and sliding semilandmarks (Cardini, 2019). In the present study, we employed both a global Procrustes alignment and sliding semilandmarks prior to conducting an analysis of integration using EMMLi (Goswami & Finarelli, 2016), but a second analysis of integration employing separate Procrustes alignments for each module returned results that were broadly consistent with, though not identical to, the first analysis. A logical next step would be to examine ontogenetic sequences of the inner ears of turkeys, which could shed light on the development of the patterns recovered in the present analysis.

## Conclusions and Future Directions

What does this all mean for turkeys, for paleontology, and for reconstructing ecology based on morphology of inner ears? First, an individual turkey’s left ear is extremely like the same turkey’s right ear. The differences between left and right ears of individual turkeys were tiny, and differences between the sample-mean left and sample-mean right were even smaller (Fig. 4). However, asymmetry appears to be a greater component of the variation in shape of avian ears—or at least, of turkey ears—than expected.

The most influential axis of shape variation in the sample seemed to be mediolateral “tilt” of rostral and caudal canals, as well as of the cochlear duct, and this was largely symmetrical variation. This could represent a “see-saw” effect whereby the most peripheral structures in the labyrinth display the largest variance in shape. Individual variation explained most of the variance in both shape and size of the inner ears in this dataset. Regardless, the Procrustes distances between left and right turkey ears were still small and differences between matching pairs of ears were not statistically clear. This suggests that avian labyrinths, or at least galliform labyrinths, are extremely similar within populations. More work is needed to assess the intraspecific variation in the morphology of the inner ears of other species of archosaurs, as different species of mammals have been shown to have different amounts of intraspecific variation in the shape of the ear (Billet et al., 2012; Perier, Lebrun & Marivaux, 2016; Gonzales, Malinzak & Kay, 2019). Still, the small amounts of variation found in this study—of a species that is not particularly aerobatic and might therefore be expected to display higher amounts of variation than more agile species (for examples of this phenomenon in mammals, see Billet et al., 2012; Perier, Lebrun & Marivaux, 2016; Gonzales, Malinzak & Kay, 2019)—suggest that higher-level inferences about populations, and possibly about species, may be confidently made when only a single set of labyrinths, or even a single right or left inner ear, is available for study.

A logical outgrowth of this line of reasoning would be to expand the sample to compare populations of wild turkeys, ultimately across the whole of the species’ range. Some populations of wild turkeys are themselves more than a million years old (Padilla-Jacobo et al., 2018), so it is entirely plausible that the pattern of variation in the shape of the ear would differ in populations of wild turkey that experience wildly different climatic and environmental regimes. Another obvious question to be answered is that if the inner ears of adult turkeys do not exhibit a clear relationship between size and shape, how does the shape of the labyrinth change across ontogeny? Studies in some species of mammals have found that the size of the inner ear does track the length of the skull through development (Ekdale, 2010) but also that the shape of the ear does not change much after becoming ossified (Jeffery & Spoor, 2004; Ekdale, 2010). Whether this pattern is also seen in the other major lineage of amniotes—the diapsids—is an open question. Finally, exploring differences between the shape of ears of wild and domesticated turkeys, in a parallel to Stange et al. (2018), would also be a natural next step to quantify the possible effects of relaxation of selective pressures under domestication.

## Supplemental Information

10.7717/peerj.7355/supp-1 Supplemental Information 1 Set of the initial 70 landmarks, in NTS format, used in the LaSEC subsampling analysis, and the graphical results of the LaSEC analysis.

10.7717/peerj.7355/supp-2 Supplemental Information 2 R code used for analyses and two 3D surface meshes of labyrinths used for visualizations within the code and article.

10.7717/peerj.7355/supp-3 Supplemental Information 3 Landmarks for each specimen, in NTS format, used throughout the analysis. Each labyrinth is represented by two replicates plus an average set of landmarks, for a total of three 35-landmark sets per labyrinth and six 35-landmark sets per specimen.

10.7717/peerj.7355/supp-4 Supplemental Information 4 Scatterplots of the first three principal components (PCs) of the variation in shape of turkey labyrinths, with points colored by specimen identity.

## Additional Information and Declarations

### Data Availability

The following information was supplied regarding data availability: 3D meshes, in PLY format, of the ears of these specimens are available in Dryad: DOI 10.5061/dryad.g76n68h. Ohio University Vertebrate Collections (OUVC) numbers are: 9803, 10218, 10231, 10239, 10443, 10599, 10610, 10628, 10657, 10736, 10739, 10869, 10884, 10885, 10888, 10890, 10891, 10892, 10893, 11403, 11406, 11407, 11408, 11410, 11411, 11413.
