# Supplementary figures and images for: Intraspecific variation and symmetry of the inner-ear labyrinth in a population of wild turkeys: implications for paleontological reconstructions
Source: PeerJ. 2019 Jul 23;7:e7355. doi: 10.7717/peerj.7355 (PMC6659666; doi:10.7717/peerj.7355)

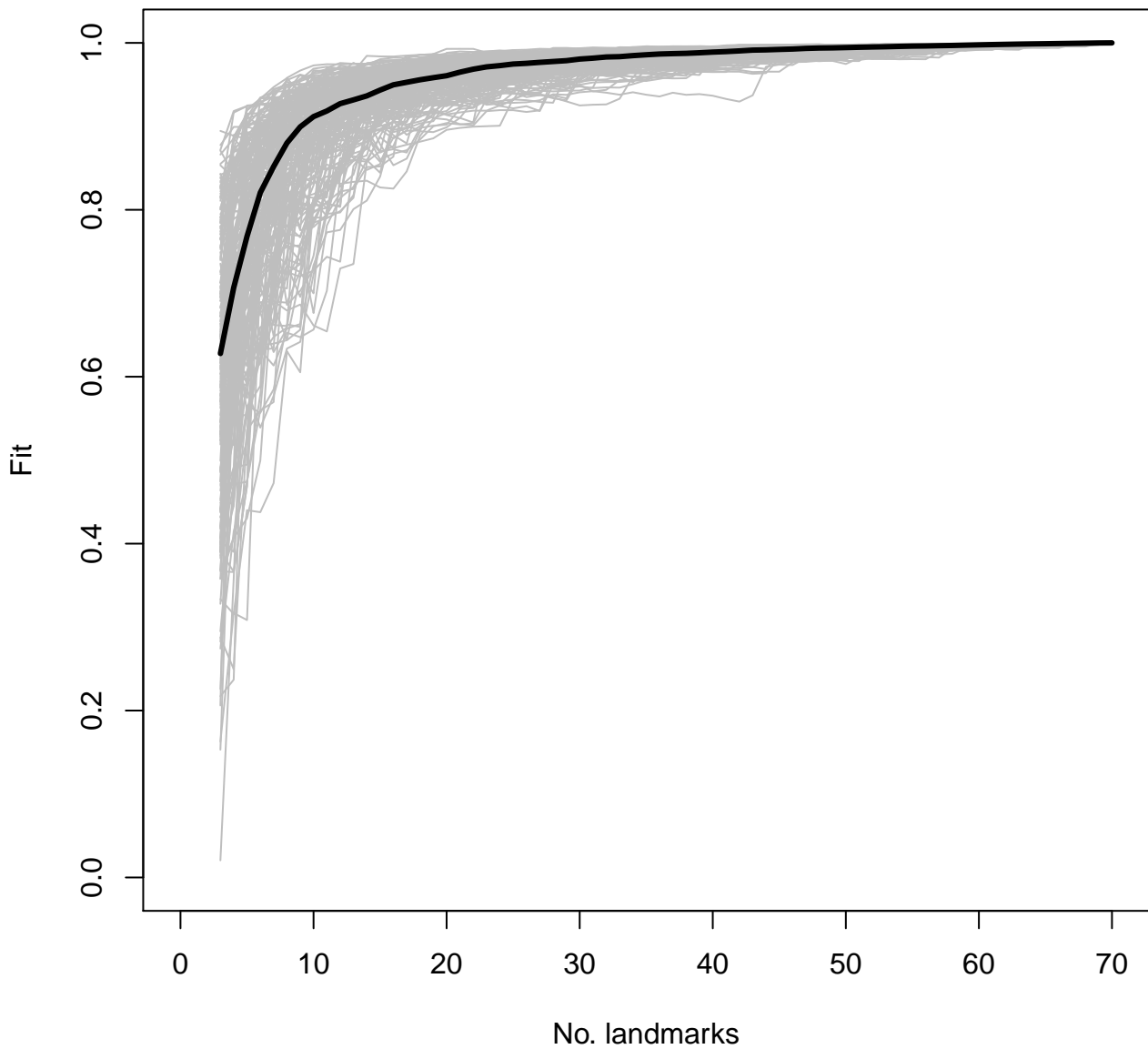

Supplement: Supplemental Information 1 [file peerj-07-7355-s001.zip › initial 70 landmarks/LaSEC_SamplingCurve_CS.pdf]

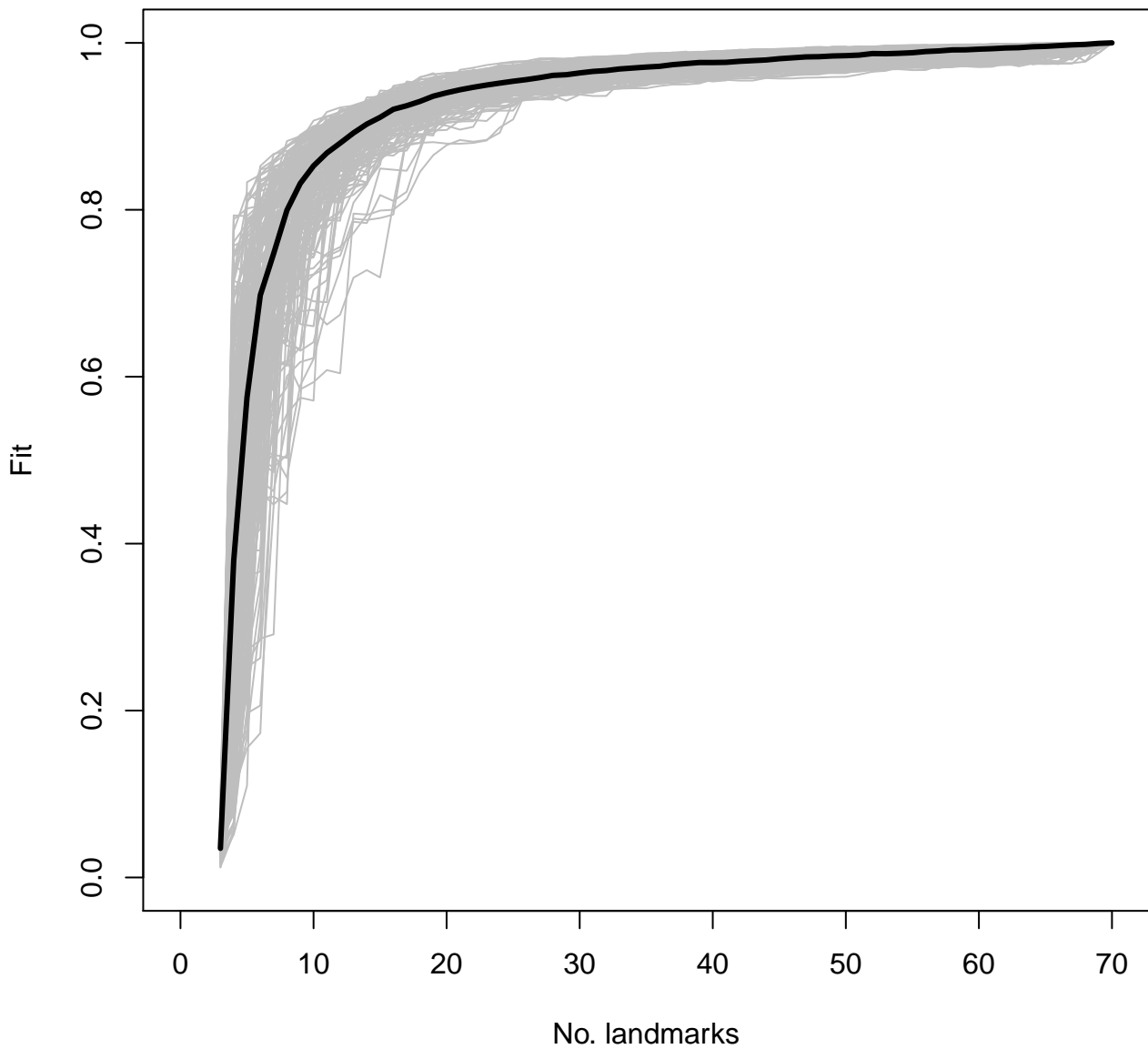

Supplement: Supplemental Information 1 [file peerj-07-7355-s001.zip › initial 70 landmarks/LaSEC_SamplingCurve_Shape.pdf]

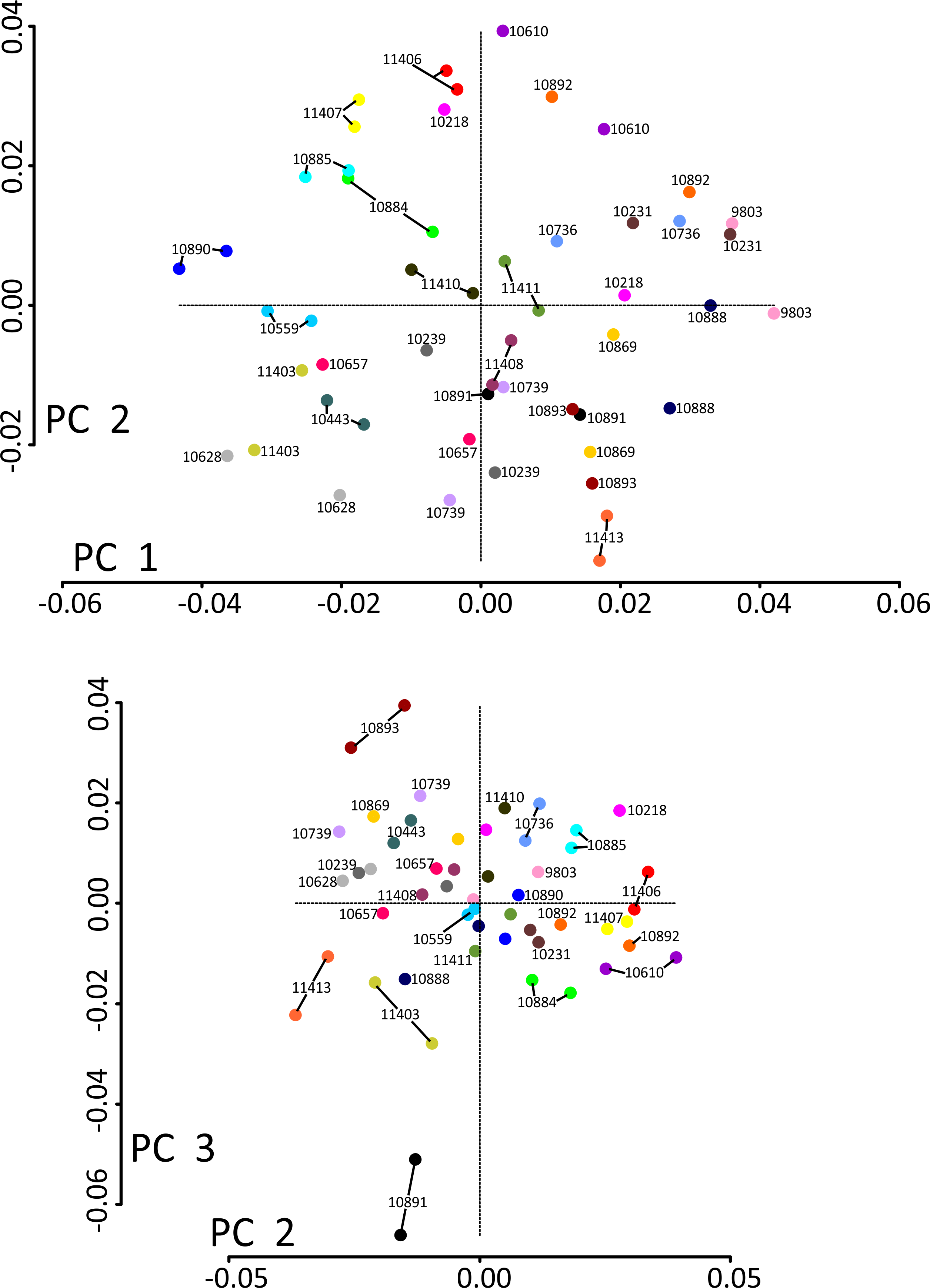

Supplement: Supplemental Information 4 [file peerj-07-7355-s004.png]
